# An Auto-luminescent Fluorescent BCG Whole Blood Assay to Enable Evaluation of Paediatric Mycobacterial Responses Using Minimal Blood Volumes

**DOI:** 10.3389/fped.2019.00151

**Published:** 2019-04-30

**Authors:** Robindra Basu Roy, Basil Sambou, Iria Uhía, Sophie Roetynck, Brian D. Robertson, Beate Kampmann

**Affiliations:** ^1^Department of Paediatrics, Centre for International Child Health, Imperial College London, London, United Kingdom; ^2^Vaccines & Immunity Theme, MRC Unit The Gambia at the London School of Hygiene and Tropical Medicine (LSHTM), Banjul, The Gambia; ^3^MRC Centre for Molecular Bacteriology and Infection, Imperial College London, London, United Kingdom; ^4^Faculty of Infectious and Tropical Diseases, The Vaccine Centre, London School of Hygiene and Tropical Medicine, London, United Kingdom

**Keywords:** tuberculosis, paediatric, BCG, immunology, mycobacterial growth inhibition assay

## Abstract

**Introduction:** Understanding protective human immunity against mycobacteria is critical to developing and evaluating new vaccines against tuberculosis. Children are the most susceptible population to infection, disease, and death from tuberculosis, but also have the strongest evidence of BCG-inducible protection. Limited amounts of blood can be obtained for research purposes in paediatrics and therefore there is a need for high-yield, low-volume, human immunology assays.

**Methods:** We transformed BCG Danish with plasmids encoding luciferase full operon derived from *Photorhabdus luminescens* together with Green Fluorescent Protein and antibiotic selection markers. We characterised the luminescent and fluorescent properties of this recombinant BCG strain (BCG-GFP-LuxFO) using a luminometer and flow cytometry and developed a paediatric whole blood *in vitro* infection model.

**Results:** Luminescence of BCG-GFP-LuxFO correlated with optical density (Spearman Rank Correlation coefficient *r* = 0.985, *p* < 0.0001) and colony forming units (CFUs) in liquid culture medium (*r* = 0.971, *p* < 0.0001). Fluorescence of BCG-GFP-LuxFO in paediatric whole blood was confirmed by flow cytometry in granulocytes and monocytes 1 h following infection. Luminescence of BCG-GFP-LuxFO in whole blood corresponded with CFUs (*r* = 0.7123, *p* < 0.0001).

**Conclusion:** The BCG-GFP-LuxFO assay requires 225 μL whole blood per sample, from which serial luminescence measurements can be obtained, together with biochemical analysis of supernatants and cellular assay applications using its fluorescent properties. This offers the opportunity to study human-mycobacterial interactions using multiple experimental modalities with only minimal blood volumes. It is therefore a valuable method for investigating paediatric immunity to tuberculosis.

## Introduction

An understanding of protective immunity to tuberculosis is required to inform vaccine design, contribute to the World Health Organization's Roadmap to end TB in children and adolescents, and achieve the goal of zero childhood tuberculosis deaths (1–6). The study of human immunity to mycobacteria is key to a better understanding of the disease, given the inherent constraints on how much can be extrapolated from animal models (7–10). Understanding paediatric immune responses is of particular importance as young children are the most susceptible to developing infection, disease, or dying following exposure, but also the group for whom there is the strongest evidence of BCG-inducible protection against tuberculosis (11–15). However, phlebotomy is technically challenging in children, can be distressing for the child and family, and research blood sample volumes are small (16). As a consequence, the number of studies conducted in children remains very limited, and a critical need to refine laboratory methodology remains. New methods need to maximise the scientific yield from small volumes of blood from children affected by *M. tuberculosis* who participate in research studies.

One widely published method to measure human mycobacterial immunity is the *lux* assay (17–27). The BCG-*lux* assay involves a 96 h incubation of whole blood in culture medium with BCG-*lux*, a strain of BCG constitutively expressing *luxAB* genes, which encode a luciferase enzyme from *Vibrio harveyii* on a plasmid together with a gene encoding hygromycin resistance to enable selection and prevent loss of the plasmid (17–27). Luminescence of samples is measured using a luminometer at baseline and 96 hours following centrifugation and red cell lysis of the pellets, dilution and addition of the luciferase substrate. The assay also provides the opportunity to store supernatants for subsequent analysis. A growth ratio is derived by the luminescence measured at 96 h divided by that measured at baseline. Another mycobacterial growth assay, the Mycobacterial Growth Inhibition Assay, measures the Time to Detection in an automated mycobacterial culture system (MGIT) of a lysed sample of whole blood or peripheral blood mononuclear cells that has been co-cultured with an inoculum of mycobacteria for 96 h (28–34).

We aimed to develop an improved *lux* whole blood assay using an auto-luminescent recombinant BCG strain expressing the luciferase full operon derived from *Photorhabdus luminescens*, which includes the genes encoding the bacterial luciferase enzyme and the genes for the synthesis of the substrate for the luminescence reaction (35–37). This would enable quantification of luminescence in a continuous manner from the same sample, and thereby decrease the blood volumes required to evaluate mycobacterial growth. Co-expression of a green fluorescent protein would enable cellular assays on cell pellets in addition to biochemical evaluation of supernatants, further enhancing the scientific yield from a given volume of blood.

## Materials and Methods

### Mycobacterial Culture Methods

BCG is a hazard group 2 organism and therefore all experimental work was conducted in category Level 2 laboratories. Liquid medium was prepared with 4.7 g of 7H9 Middlebrook dried broth (Fluka/Sigma-Aldrich, Gillingham, UK), 2.5 ml of 20% Tween 80 (Acros Organics/Thermo Fisher Scientific, Waltham, MA, USA), 4 ml of 50% glycerol (VWR, Radnor, PA, USA), and dissolved in 893.5 ml of dH_2_O. This was then autoclaved for 15 min at 100 kPa at 1210°C. Once cooled 100 ml of Albumin Dextrose Catalase (ADC) supplement (BD, Franklin Lakes, NJ, USA) was added and selection antibiotics at the specified concentrations (Hygromycin, Sigma-Aldrich, Gillingham, UK; Kanamycin, Glyco/Life Technologies, Carlsbad, CA, USA). Forty microlitre of 20% Tween 80 was added for each 15 ml of medium. Solid medium was prepared with 21 g of 7H11 agar (Fluka/Sigma-Aldrich, Gillingham, UK) and 10 ml 50% glycerol made up to a final volume of 900 ml with dH_2_O in a 1 L bottle and autoclaved for 15 min at 100 kPa at 1210°C. Once cooled, 100 ml of Oleic acid Albumin Dextrose Catalase (OADC) supplement (BD, Franklin Lakes, NJ, USA) was added and selection antibiotics at the specified concentrations. Colony Forming Units (CFU) were counted using serial dilutions in sterile Phosphate Buffered Saline of 10 μL of the experimental sample plated onto solid culture medium after 21 days. Liquid and solid cultures were incubated at 37°C. Optical density (OD_600_) of 0.5 ml volumes of bacteria in liquid culture was measured using a Biophotometer Plus spectrophotometer (Eppendorf, Hamburg, Germany).

### Transformation of BCG

The parent strain was BCG Danish (Staten Serum Institute, Copenhagen, Denmark). The following plasmids were used: pMV306DIhsp+LuxG13: Kanamycin resistance integrase-free integrative reporter plasmid harbouring the Luciferase Full Operon (*Photorhabdus luminescens*) (35, 37, 38). (pMV306DIhsp+LuxG13, Addgene plasmid # 49999) (37); pBS-Int: a suicide plasmid that expresses the integrase gene necessary for the integration of pMV306DIhsp+LuxG13 into the chromosome after electroporation. (Addgene plasmid # 50000); pGFPHYG2: A hygromycin resistance replicative plasmid that expresses gfpmut3, encoding Green Fluorescent Protein, and (39) (pGFPHYG2, Addgene plasmid # 30173).

Transformations were carried out as per the protocol of Goude and Parish (40) Briefly, 50 mL of culture in logarithmic growth phase were centrifuged at 3000 × g for 10 min at room temperature. The cells were washed twice with 50 ml and then 20 ml pre-warmed 10% glycerol plus 0.05% Tween 80. The cells were resuspended in 500 μL of 10% glycerol and 200 μL was transferred into electroporation cuvettes. One hundred nanograms DNA of each plasmid was used. Electroporation was carried out with a single pulse of 2.5 kV, 25 μF with the pulse-controller resistance set at 1,000 Ω resistance. The suspension was then immediately transferred into 10 mL 7H9 media and incubated without antibiotics for 16 h. Bacteria were harvested by centrifugation at 3000 × g for 10 min and dilutions were plated out on 7H11 plates with kanamycin at 20 μg/mL and hygromycin at 50 μg/mL. Plates were incubated at 37°C until discrete colonies appeared. Colonies were picked and incubated in 7H9 liquid media at 37°C. Stocks were frozen down in 15% glycerol. The resultant strain is named BCG-GFP-LuxFO.

### Quantification of Luminescence and Detection of Fluorescence of Bacteria

Luminescence of BCG-GFP-LuxFO was quantified in Relative Light Units/second (RLU/s) using the Sirius Tube Luminometer (Berthold Detection Systems GmbH, Pforzheim, Germany) immediately following removal from the incubator.

Direct fluorescence of bacteria was measured in a 1:1 mixture of non-fluorescent BCG and BCG-GFP-LuxFO fixed in 2% paraformaldehyde using a LSRII Fortessa flow cytometer (BD, San Jose, CA, USA).

### Paediatric Whole Blood BCG-GFP-LuxFO Assays

Whole blood was collected in lithium-heparin vacutainer tubes (BD, Wokingham, UK) from children aged 5–15 years old participating in a household contact tuberculosis study in The Gambia (41–44). Each independent experiment consisted of participants who were selected based upon having had household exposure to the same adult with smear-positive pulmonary tuberculosis, no symptoms of tuberculosis, and included children with positive and negative tuberculin skin test responses as part of a larger study exploring factors influencing latent tuberculosis infection in children. Approvals were obtained from The MRC Unit The Gambia Scientific Coordinating Committee and The Gambia Government/MRC Joint Ethics Committee (SCC1405). Experiments were carried out in 2015 at MRC Unit The Gambia at the London School of Hygiene and Tropical Medicine. Participant characteristics are shown in Table 1.

**Table 1:** Demographic characteristics of 9 participants.

Median age in years (interquartile range)	7.3 (6.5–8.5)
Male:Female ratio	1:2
TST result>10 mm	3

A vial of BCG-GFP-LuxFO stored in 15% glycerol at −70°C was thawed and added to 15 ml 7H9 Middlebrook broth supplemented with ADC and kanamycin (final concentration 20 μg/ml) and hygromycin (final concentration 50 μg/ml) +40 μl 20% Tween 80 in a 250 ml Erlenmeyer flask (Corning B.V Life Sciences, Amsterdam, The Netherlands). This was incubated in an orbital shaking incubator at 37°C with daily measurements of 0.5 ml samples for luminescence and optical density. Further liquid medium was added as required to maintain the bacteria in logarithmic growth phase until the day of the assay. Immediately prior to the blood samples being taken, an aliquot of the stock in logarithmic phase was diluted down to 3.3 x 10^5^ RLU/ml/s BCG-GFP-LuxFO. This concentration of BCG-GFP-LuxFO gave baseline luminescence readings of whole blood samples 10-fold higher than the experimentally determined detection threshold of the luminometer used for this assay to enable a dynamic range.

**Figure 1:**
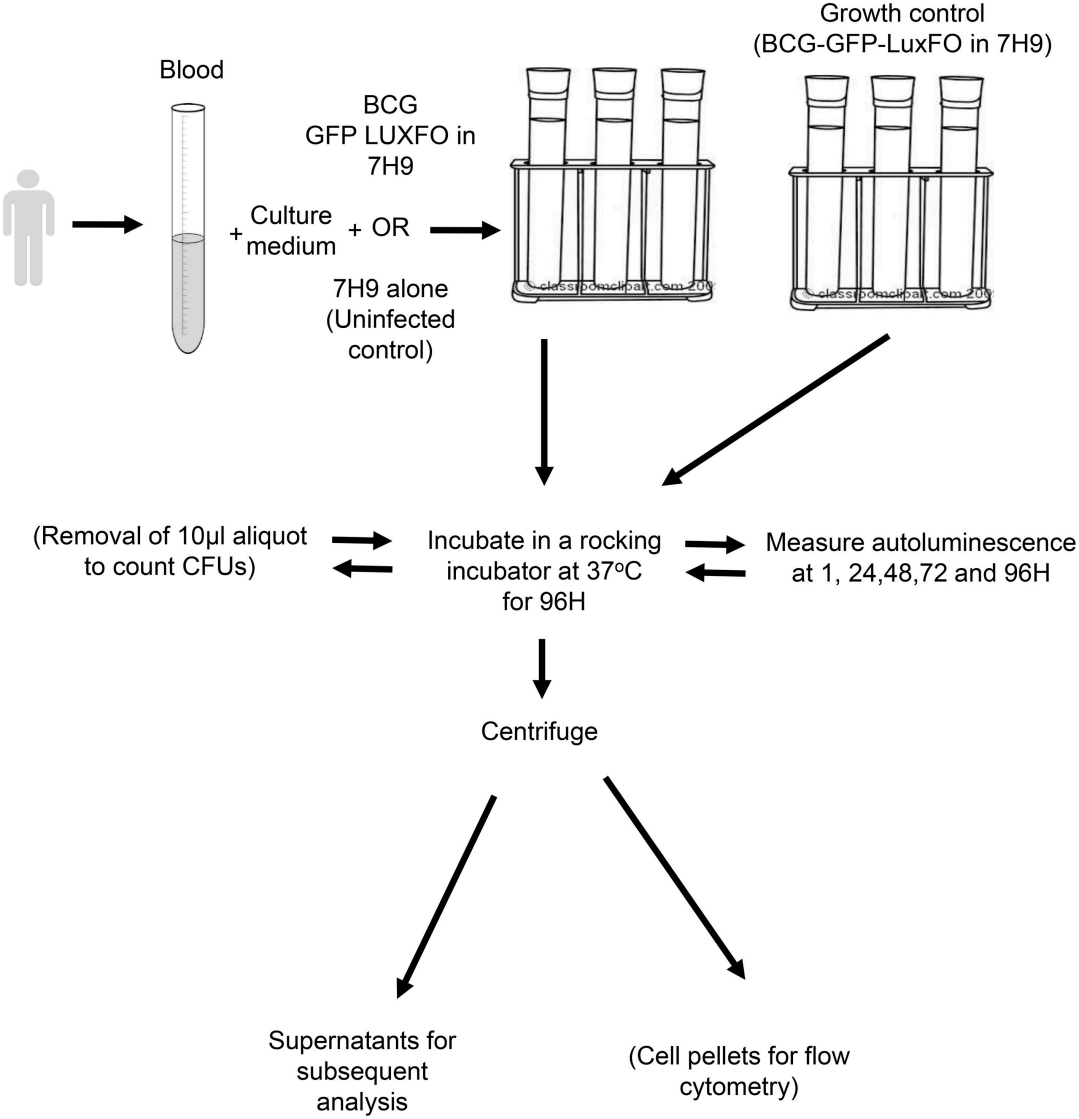
Schematic of BCG-GFP-LuxFO whole blood assay method.

A schematic of the paediatric whole blood assay is shown in Figure 1. Whole blood was mixed in a ratio of 1:1 with RPMI 1640 culture medium (Sigma-Aldrich, Gillingham, UK) containing 2.5% 1M HEPES buffer and 1% L-glutamine (both Sigma-Aldrich). The BCG-GFP-LuxFO in 7H9–0.05% Tween 80-ADC with kanamycin at 20 μg/mL and hygromycin at 50 μg/mL was added at a ratio of 1 part BCG-GFP-LuxFO: 9 parts whole blood in culture medium. For the medium only control samples, bacterial medium alone was added in the same ratio of 1:9. Four 0.5 ml aliquots of each experimental condition were placed into sterile lidded 75 × 12 mm standard tubes (Corning B.V Life Sciences, Amsterdam, The Netherlands). This equates to a total volume of 900 μL whole blood for each condition with triplicate tubes for quantifying luminescence and a fourth tube to enable determination of CFU during this method development. Triplicate growth controls of the recently diluted BCG-GFP-LuxFO in 1:9 ratio with further liquid medium were prepared in parallel to confirm that the bacteria remained in logarithmic growth phase at the time of inoculation.

### Detection of Fluorescence in Paediatric Whole Blood

One hour after BCG-GFP-LuxFO or medium alone were added, samples were centrifuged at 2,000 g for 10 min. The cell pellet was resuspended in RPMI with 2 mM EDTA to remove adherent cells, and red cells were then lysed with a 10 min incubation with red cell lysis buffer (eBioscience, Thermo Fisher Scientific, Waltham, MA, USA), washed, a further 10 min incubation with red cell lysis buffer and then washed twice. 0.4 μL Live/Dead Aqua (Thermo Fisher Scientific, Waltham, MA, USA) was added and incubated for 20 min in the dark at room temperature. Cells were then washed and fixed in FACS Lysing solution for 10 min (BD, San Jose, CA, USA), washed twice and resuspended in PBS with 2% Fetal Calf Serum and 0.5 mM EDTA. Data were acquired with a LSRII Flow Cytometer (BD, San Jose, CA, USA). Cells were first gated based on their light scatter properties and cellular debris were excluded. Only singlets and live cells were then included in the analysis. Compensation was calculated for each fluorescence using single colour stained whole blood samples (i.e., medium only control sample stained with Live/Dead dye and unstained BCG-GFP-LuxFO infected sample). Data analysis was performed using FlowJo v10.5.3 for Mac (Treestar Inc., USA).

### Detection of Luminescence in Paediatric Whole Blood

Luminescence of each whole blood aliquot was measured using the Sirius Tube Luminometer (Berthold Detection Systems GmbH, Pforzheim, Germany) immediately following removal of the tubes from the incubator. Luminescence was measured 1 h after the bacteria were added to ensure that the sample had equilibrated to 37°C in the incubator, and at 24, 48, 72, and 96 h. Three luminescence measurements at each time point were recorded, and the mean of these three measurements was recorded. At each time point, mean background luminescence from three medium-only control samples was also measured and then subtracted. Ten microlitre aliquots were removed from a fourth tube every 24 h and dilutions were plated for determination of CFUs and correlation to the luminescence measurements in the same tube.

### Statistical Methods

Spearman's Rank Correlation coefficients and coefficients of variation were calculated using Prism 7 for MacOS X (Graphpad Software, La Jolla, CA, USA). Two-tailed significance testing was applied with *p* < 0.05 considered to be statistically significant.

## Results

### Characteristics of BCG-GFP-LuxFO in Liquid Culture

**Figure 2:**
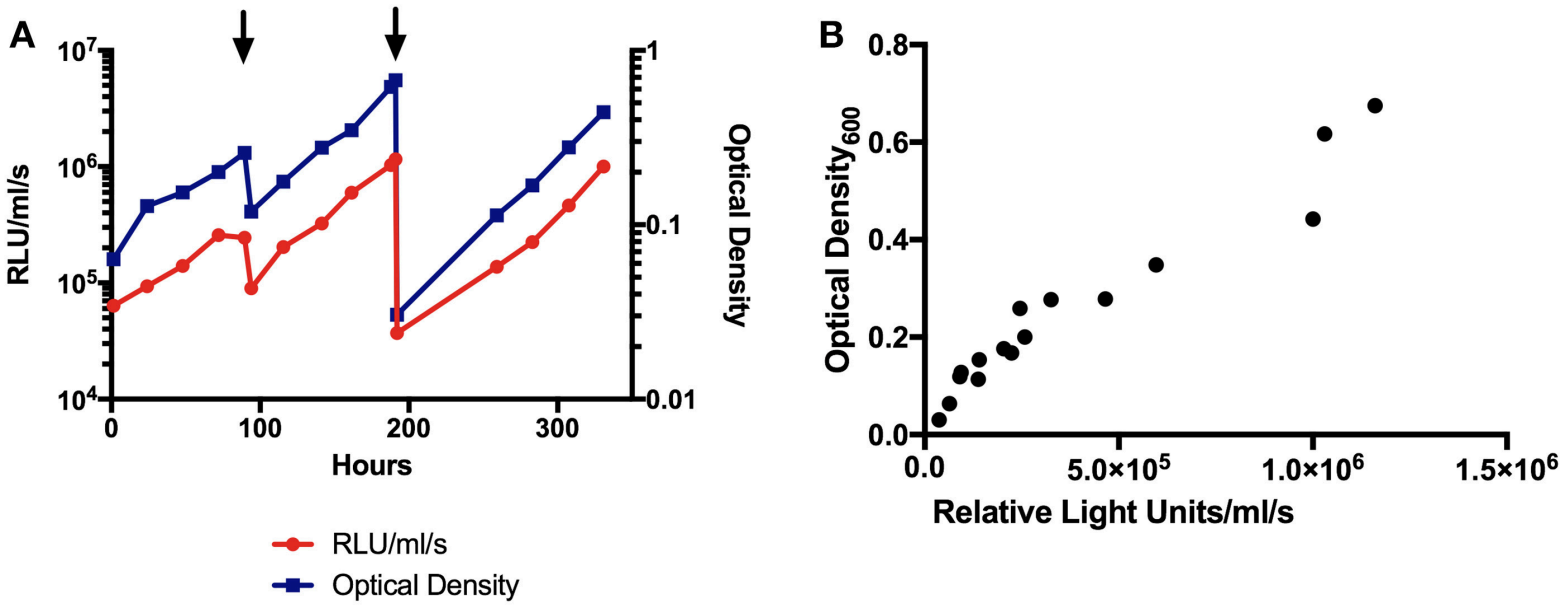
**(A)** Luminescence and optical density growth curves of BCG-GFP-LuxFO in liquid culture. Luminescence is shown on the left y axis, indicated in red, and optical density on the right y axis, indicated in blue. Arrowheads denote addition of media to maintain bacteria in logarithmic growth phase. **(B)** Correlation between optical density and luminescence measurements of BCG-GFP-LuxFO in 7H9 liquid medium. *n* = 16 pairs of observations. Spearman rank correlation coefficient, *r* = 0.9853 (95% CI:0.9556:0.9952; *p* < 0.0001). RLU, Relative Light Units.

**Figure 3:**
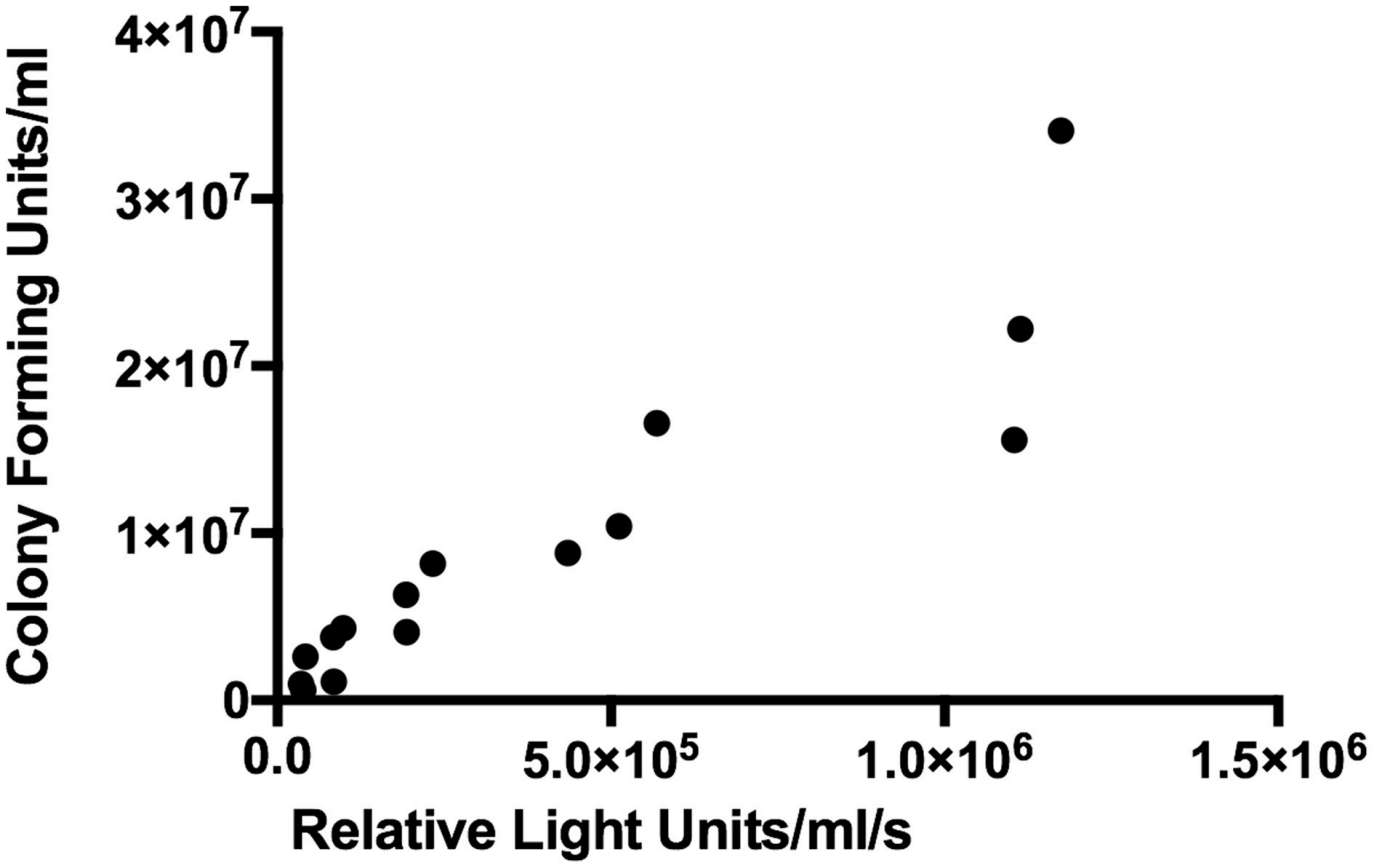
Correlation between colony forming units and luminescence of BCG-GFP-LuxFO in liquid 7H9 medium. *n* = 15 pairs of observations. Spearman rank correlation coefficient *r* = 0.9714 (95% CI: 0.9112–0.9910; *p* < 0.0001).

**Figure 4:**
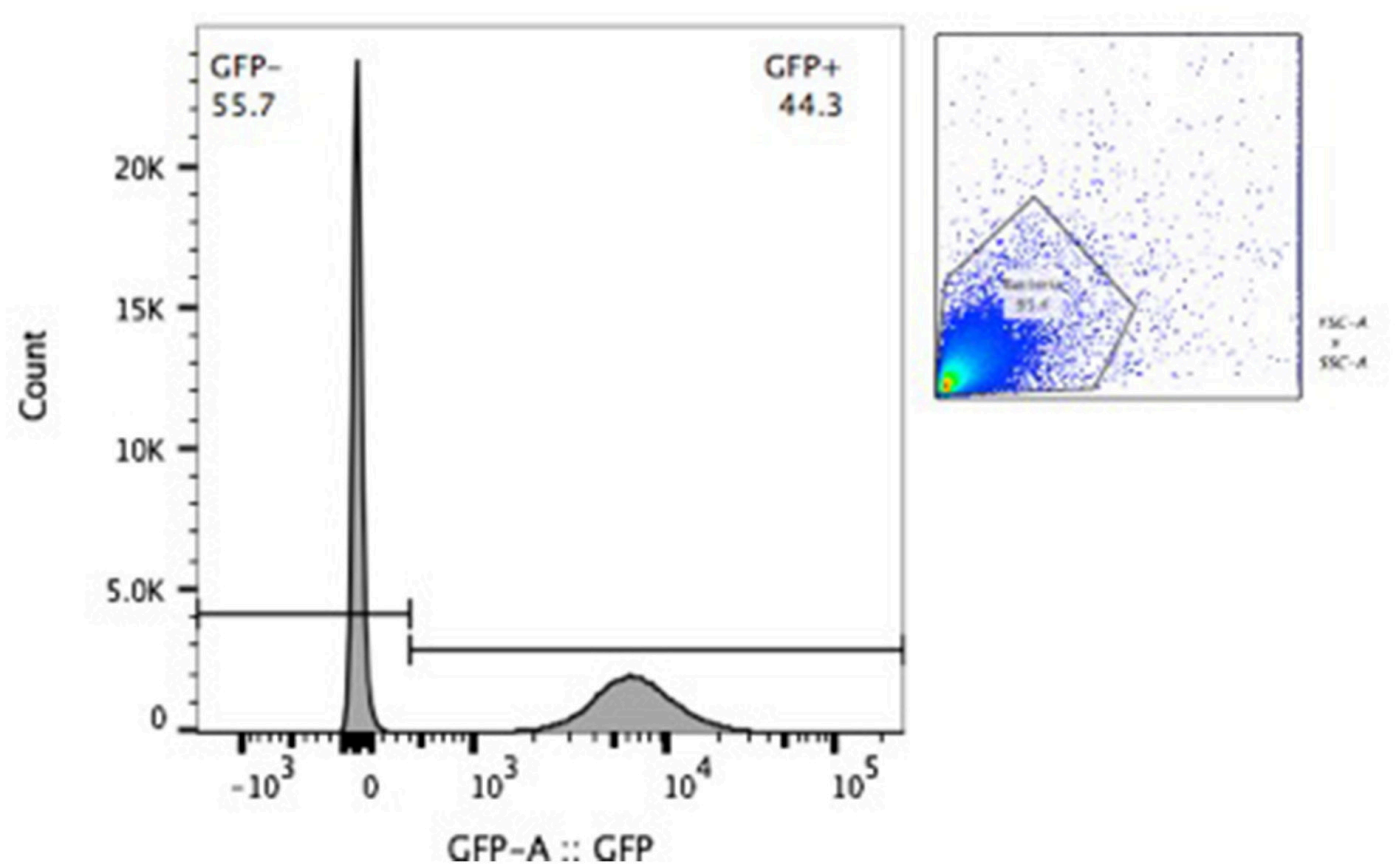
Flow cytometric confirmation of fluorescence of BCG-GFP-LuxFO in a 1:1 mixture of non-fluorescent BCG and BCG-GFP-LuxFO.

BCG-GFP-LuxFO demonstrated logarithmic growth in liquid culture medium (Figure 2A) with luminescence and optical density correlating with Spearman Rank Correlation coefficient of 0.985 (95% confidence interval 0.956–0995, *p* < 0.0001) (Figure 2B). Colony forming units and luminescence of BCG-GFP-LuxFO in liquid culture medium correlated with Spearman rank correlation coefficient *r* = 0.9714 (95% CI: 0.9112–0.9910; *p* < 0.0001) (Figure 3). The RLU: CFU ratio for BCG-GFP-LuxFO was 0.05 RLU: 1 CFU. Fluorescence of BGC-GFP-LuxFO was confirmed by flow cytometry (Figure 4).

### Fluorescence in Paediatric Whole Blood Assay

**Figure 5:**
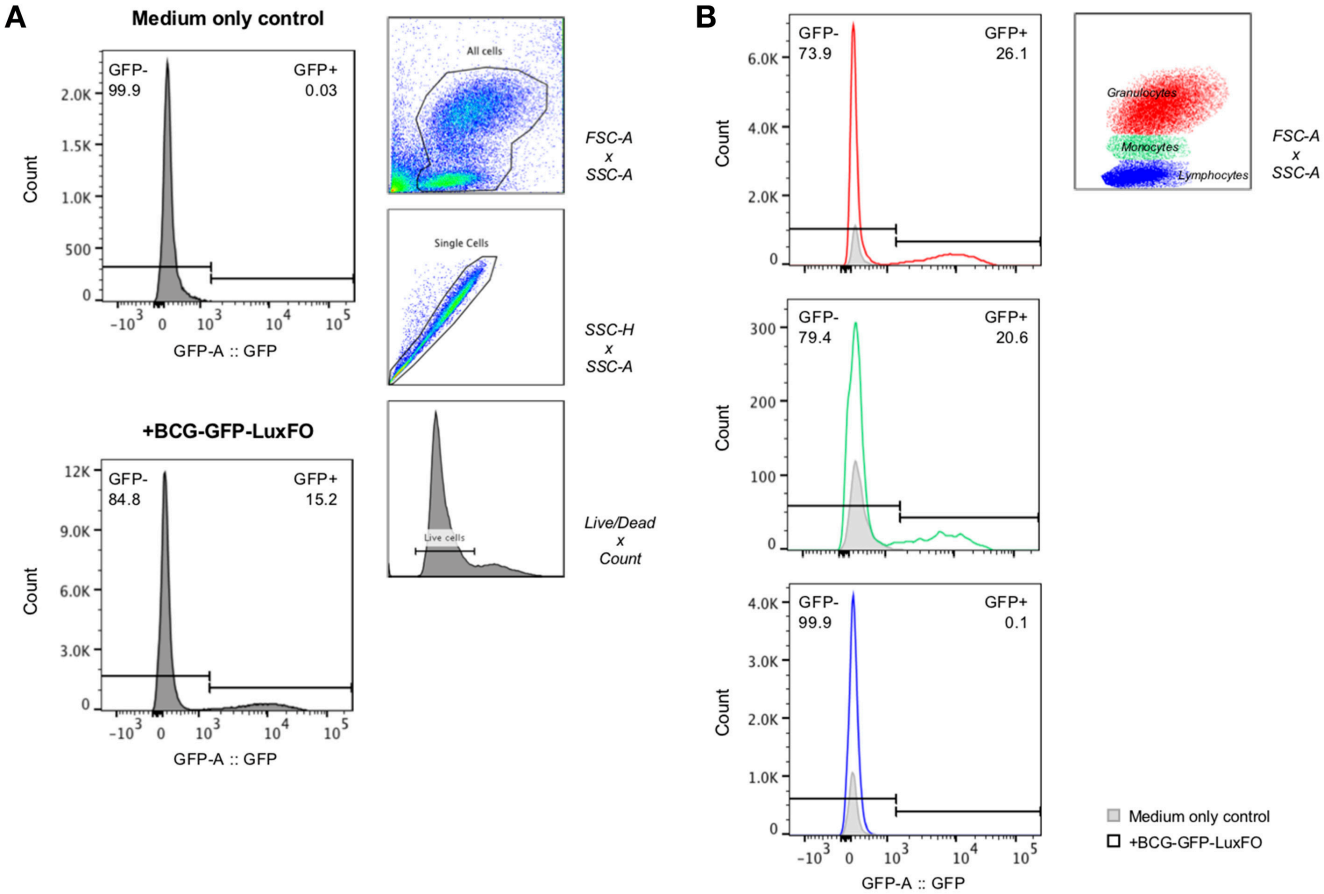
Flow cytometric detection of fluorescence of BCG-GFP-LuxFO in whole blood assay. **(A)** Within the live cell population, GFP– and GFP+ cells were determined, respectively. **(B)** Within the live cell population, cells were then gated on either granulocytes (in red), monocytes (in green), or lymphocytes (in blue), distinguishable by their size and granularity using forward scatter (FSC)/side scatter (SSC) gate. The gate GFP–/GFP+ populations were set based on the medium only negative control so that the frequency of GFP+ cells = 0% for each cell population. For each plot, the GFP- population is reported in the top left, and the GFP+ population in the top right. Representative data shown from samples from two children one hour after BCG-GFP-LuxFO was added.

Following centrifugation to obtain the cell pellet, 350–400 μL of supernatant could be stored from each 0.5 ml aliquot. One hour after infection with BCG-GFP-LuxFO, 15% of all cells were GFP positive, with 26% of granulocytes, 21% of monocytes, and 0.1% of lymphocytes demonstrating fluorescence based on cell size and granularity (Figure 5).

### Luminescence in Paediatric Whole Blood Assay

**Figure 6:**
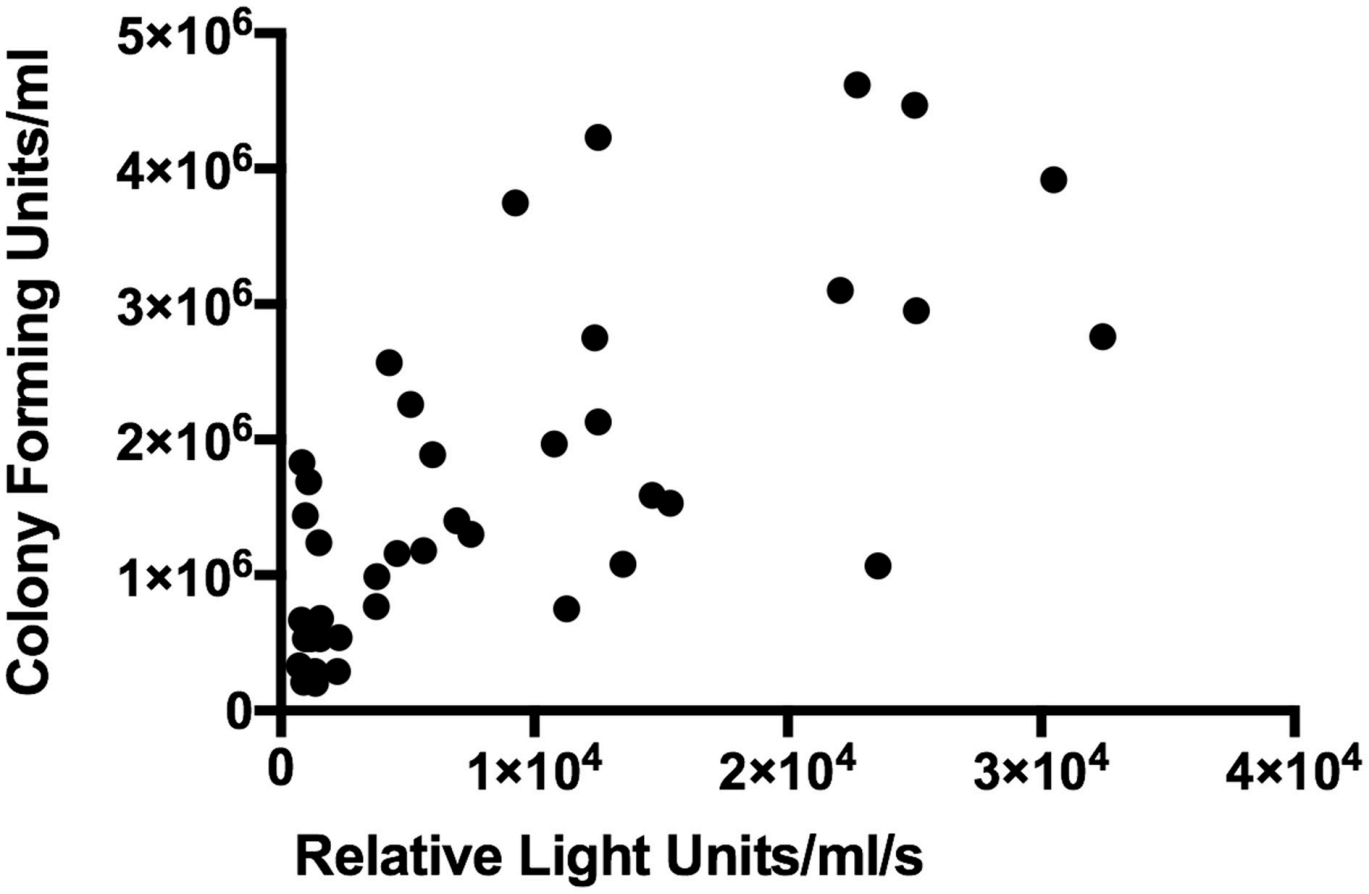
Correlation between luminescence and colony forming units in BCG-GFP-LuxFO paediatric whole blood assay. *n* = 45 pairs of observations (samples from three experiments, from 9 children at 5 time points). Spearman rank correlation coefficient *r* = 0.7123 (95%CI: 0.5230–0.8346; *p* < 0.0001).

Data were analysed from nine children in three independent experiments and five timepoints. Luminescence and CFUs correlated with Spearman rank correlation coefficient *r* = 0.7123 (95%CI: 0.5230–0.8346; *p* < 0.0001) (Figure 6). Mean coefficients of variation in luminescence data across these experiments were 7.65% for (a) the three measurements of each tube and 11.5% for (b) the three replicates for each child.

## Discussion

We have developed and evaluated an auto-luminescent fluorescent BCG reporter strain that demonstrates robust correlations between luminescence, optical density, and CFUs in liquid culture. In a high TB-endemicity setting with paediatric whole blood samples, we were able to demonstrate across three independent experiments, nine paediatric subjects, and five timepoints that luminescence and CFU correlated significantly. The BCG-GFP-LuxFO assay enables serial non-destructive luminescence measurements over 96 h where each tube contains 225 μL whole blood. Existing assays require 450 μL to generate a single 96 h growth ratio value in the *lux* assay or 300 μL of whole blood to generate the Time to Detection in the Mycobacterial Growth Inhibition Assay (23, 32, 34). The ability to quantify luminescence in a non-destructive manner combined with the fluorescent nature of BCG-GFP-LuxFO means that following measurement, samples can be centrifuged to carry out biochemical analyses on the supernatants and cellular analyses on the cell pellet as required.

BCG-GFP-LuxFO has a relatively low RLU:CFU of 0.05 RLU: 1 CFU, which likely reflects the associated metabolic costs of constitutive expression of the whole luciferase operon (35–37). This low RLU:CFU ratio means that the multiplicity of infection is higher than with the *lux* assay. However, as measuring luminescence in the original *lux* assay involves lysis of red blood cells, vortexing with glass beads, and dilution steps, there is considerable decrease in experimental time with the BCG-GFP-LuxFO assay compared to the *lux* assay, and the potential for automated luminescence measurement (20).

The BCG-GFP-LuxFO assay contributes to the range of immunological methods available to study human whole blood responses to mycobacteria. Studies of human whole blood have made many important contributions to understanding human mycobacterial immunity and originally led to the identification of mendelian susceptibility genes and pathways (17, 18, 27, 45, 46). Studying whole blood in paediatric populations with tuberculosis has highlighted distinct gene expression signatures with diagnostic potential (47). In light of recent advances in the immunological definition of the TB spectrum, the BCG-GFP-LuxFO assay has potential to compliment other research methods in characterising the human host response (8, 46, 48–50).

Whole blood is critical to study the role of neutrophils in tuberculosis: they are known to be the most common cell type infected in whole blood samples taken from adult donors (51); enhance macrophage responses (52); and drive a type I interferon-inducible transcript signature in adults (45). The small volume requirements for the BCG-GFP-LuxFO assay combined with the high data yield means that it is well-suited to research in paediatric populations affected by tuberculosis.

## Ethics Statement

This study was carried out in accordance with the recommendations of The MRC Unit The Gambia Scientific Coordinating Committee and The Gambia Government/MRC Joint Ethics Committee (Reference number: SCC1405), with written informed consent from all families. All subjects gave written informed consent in accordance with the Declaration of Helsinki.

## Author Contributions

RB, IU, BK, and BR conceived and designed the work. IU and RB carried out the transformations of BCG. RB and BS developed and carried out the whole blood assays. SR and RB carried out the flow cytometry experiments and analysis. RB drafted the work and all authors revised it critically for important intellectual content and are accountable for all aspects of the work.

### Conflict of Interest Statement

BK holds a patent for a paediatric diagnostic biosignature. RB was a consultant for FIND, Geneva, a non-profit organization, from 2014 to 2016. The remaining authors declare that the research was conducted in the absence of any commercial or financial relationships that could be construed as a potential conflict of interest.
